# Development of Chinese mental health first aid guidelines for assisting a person affected by a traumatic event: a Delphi expert consensus study

**DOI:** 10.1186/s12888-021-03606-3

**Published:** 2021-12-01

**Authors:** Yan Wang, Wenjing Li, Shurong Lu, Anthony F. Jorm, Brian Oldenburg, Yanling He, Nicola Reavley

**Affiliations:** 1 Shanghai Mental Health Centre, Shanghai Jiao Tong University School of Medicine, 600 Wan Ping Nan Road, Shanghai, 200030 China; 2 Centre for Mental Health, Melbourne School of Population and Global Health, University of Melbourne, Victoria, Australia; 3 Jiangsu Provincial Centre for Disease Control and Prevention, Nanjing, China; 4 The Nossal Institute for Global Health, Melbourne School of Population and Global Health, University of Melbourne, Victoria, Australia

**Keywords:** Mental health first aid, Trauma, Cultural adaptation, Delphi study, China

## Abstract

**Background:**

People who experience traumatic events have an increased risk of developing a range of mental disorders. Appropriate early support from people in a person’s social network may help to prevent the onset of a mental disorder or minimize its severity. Mental health first aid guidelines for assisting people who have experienced traumatic events have been developed for high-income English-speaking countries. However, they may not be appropriate for use in China due to cultural and health care system differences. The aim of this study was to develop culturally appropriate guidelines for people providing mental health first aid to people affected by traumatic events in China.

**Methods:**

A Delphi expert consensus study was conducted with two panels of experts in mainland China. Experts recruited to the panels included 32 professionals with expertise in the treatment of people affected by traumatic events and 31 people with lived experience of trauma or their carers. Panel members were sent a Chinese translation of the questionnaire used for developing English-language mental health first aid guidelines. This contained 168 items describing how to help people experiencing a potentially traumatic event. Panelists were asked to rate the importance of each statement for inclusion in the Chinese guidelines. They were also encouraged to suggest any additional statements that were not included in the original questionnaire. Statements were accepted for inclusion in the adapted guidelines if they were endorsed by at least 80% of each panel as very important or important.

**Results:**

Consensus was achieved after three survey rounds on 134 statements for inclusion in the adapted guidelines for China, with 127 adopted from the guidelines for English-speaking countries and 7 new items from the comments of panelists.

**Conclusions:**

While many of the statements are similar to the guidelines for English-speaking countries, the panelists adapted the guidelines to China’s context, including more detailed actions on how to discuss trauma and to help the person. These guidelines will be used to form the basis of a Mental Health First Aid (MHFA) training course for China, aimed at educating the public in providing support and advice to a person who is experiencing a potentially traumatic event. Further research is needed to investigate the use of the guidelines by the Chinese public and the implementation of MHFA training in appropriate settings in China.

**Supplementary Information:**

The online version contains supplementary material available at 10.1186/s12888-021-03606-3.

## Background

Traumatic events are those that may cause powerful and distressing experiences that are usually life threatening or pose a significant threat to a person’s physical or psychological wellbeing. They include accidents, physical assaults, and natural disasters. After a traumatic event, without timely intervention, people are at significantly increased risk of experiencing anxiety, depression, insomnia, self-injury and other psychological and behavioral problems, and may even develop an acute stress disorder (ASD), post-traumatic stress disorder (PTSD) or other mental illness [1, 2]. A number of studies in China have examined the impact of traumatic events on mental health problems. These include the 2008 Wenchuan earthquake, after which the prevalence of PTSD was estimated to be 22.1% at 8 months, 19.8% at 14 months, 19.0% at 26 months, and 8.0% at about 44 months [3]. During the coronavirus (COVID-19) epidemic in China between late 2019 and early 2020, an online survey open to the public showed that almost 35% of the respondents experienced mental distress [4]. Another study investigated the mental health characteristics of 53,427 people during the epidemic, and the results showed a high prevalence of anxiety symptoms (97.75%), depressive symptoms (97.48%), insomnia symptoms (69.25%) and symptoms of PTSD (4.75%) among the general public [5].

When a potentially traumatic event occurs, timely and appropriate help from friends, family members or colleagues can reduce a person’s risk of developing a mental health problem or reduce its severity [6–8]. However, the majority of people do not have the knowledge, skills or confidence required to assist a person developing a problem or in a mental health crisis [9]. In China, many people do not access medical treatment after potentially traumatic events, due to limited availability of services and also because of poor mental health literacy or stigma [10, 11]. In order to enhance the public’s mental health knowledge, self-help and helping skills, the Chinese government has issued several policy documents in recent years, proposing to improve the public’s mental health literacy, strengthen the delivery of mental health care, and encourage the promotion of awareness and approaches to potentially traumatic events [12, 13].

With the context of growing awareness of the impact of traumatic events on mental health in China, experts have developed a number of psychological first aid guidelines. Most of these guidelines have been translated directly from the English versions developed for use in other countries or are targeted towards health professionals. For example, in the immediate aftermath of the Wenchuan earthquake in 2008, Chinese experts organized the translation of “Psychological First Aid: Field Operations Guide, 2nd Edition” from the National Child Traumatic Stress Network and National Center for PTSD of the United States [14]. In addition, the World Health Organization published the “Psychological first aid: Guide for field workers” [15]. These guidelines are primarily provided to mental health workers or volunteers and some of the actions recommended in these guidelines are not appropriate for the general public. Some were developed for use in the aftermath of specific events and the effectiveness of these guidelines has not been systematically evaluated in the context of Chinese culture.

Mental health first aid has been defined as “the help provided to a person who is developing a mental health problem, experiencing a worsening of an existing mental health problem or in a mental health crisis; the first aid is given until appropriate professional help is received or the crisis resolves” [16]. The Mental Health First Aid (MHFA) training program was established in Australia in 2000 in response to the need for public education about mental illness and its treatment. Primarily designed for non-health professionals, the training has since spread to every state and territory in Australia and is currently available in 27 countries. By 2020, over 850,000 Australians and almost 4 million people worldwide had attended an MHFA course [17]. Many previous studies have shown that MHFA training is associated with improved recognition of mental disorders, mental health first aid knowledge, beliefs about effective treatments, confidence and intentions to provide assistance to individuals with mental health problems, and amount of help provided, and also with decreases in stigmatizing attitudes [18–21]. Some studies have also shown improved mental health in those who attend the training [21–23]. Three studies of Chinese communities in Australia and Hong Kong have also shown similar effects [24–26].

The MHFA training course was based on a series of guideline documents that were developed using the Delphi method, which is a systematic way of assessing the consensus of a panel of experts [27]. The guidelines contain information on a range of strategies to help people developing mental health problems (e.g. depression, psychosis) or in a mental health crisis (e.g. suicide). They include mental health first aid guidelines for supporting a person affected by a traumatic event, which were originally conducted in 2008 and updated in 2020. However, most of the guideline development studies have been done in high-income English-speaking countries [28–30], with only a few, on how to assist a person at risk of suicide, carried out in middle-income countries, including the Philippines, Sri Lanka, India and China [31–34]. The research team have previously conducted studies to develop guidelines on providing mental health first aid to a Chinese person developing depression, psychosis, problem drinking or at risk of suicide [34–37]. No cultural adaptation studies of trauma-related guidelines have been conducted. China’s cultural background and mental health system and policies are quite different from those of high-income English-speaking countries. Whether trauma guidelines for English-speaking countries would apply to Chinese culture and health system is unclear.

The aim of this study was to develop culturally appropriate guidelines for people providing assistance to a person after a potentially traumatic event in China. Through the promotion and application of the guidelines, we hope to promote public awareness of traumatic events and their impact, and to improve the skills of people supporting others in the aftermath of traumatic events among the general public in China.

## Methods

### The Delphi method

We used the Delphi method to reach consensus on the inclusion of guideline statements. Generally, the Delphi method involves a number of iterations before consensus is achieved. Feedback is given at each round in order to help experts compare their opinions to those of the group. This method has been used in many countries to develop mental health first aid guidelines for a wide range of mental health problems or crises, including depression, psychosis and alcohol problems [38].

This Delphi consensus study involved four steps: (1) questionnaire development, (2) panel recruitment and formation, (3) data collection and analysis, and (4) guidelines development.

### Questionnaire development

First, the Round 1 questionnaire from the redevelopment of the English-language guidelines [39] was translated into Chinese by a professional translator. Then the Chinese working group (YW, YH, WL and SL) checked the Chinese-language version and made several rounds of modifications to the statements and structure of the questionnaire to ensure that it was appropriate for the culture and health service systems in China. All working group members are skilled in both English and Chinese languages and are familiar with mental health system in China.

A total of 168 statements were included in the first round of the questionnaire and they were divided into 6 sections according to the content of the statements, including: (1) Background information, (2) Actions to be taken at the site of a potentially traumatic event, (3) Talking about the trauma, (4) Providing support in the weeks and months following a potentially traumatic event, (5) Experiences of abuse, and (6) Adolescents.

The questionnaire started with informed consent and questions about the demographic characteristics of the participants. This survey was conducted online using a Chinese survey tool, Questionnaire Star. If someone did not agree to participate in the survey, he/she could opt out of the survey page. The survey only continued if he/she chose the “agree” option.

### Panel recruitment and formation

Participants were recruited into one of the two panels: professionals and people with lived experience (either as consumers or carers). Members of the professional panel could be a psychiatrist, psychotherapist, counselor, or a school teacher who was accredited to provide psychological counseling to students. They were required to have some knowledge about trauma issues and to have at least 2 years of occupational experience in mental health related to trauma. Professionals were approached via publicly available contact details or through personal contacts of the Chinese research team. Participants were also encouraged to pass on the invitation to other eligible participants.

Members of the lived experience panel were people who had personally experienced traumatic events, patients with PTSD, or their carers (e.g., volunteers who care for families who have lost their only child). Potential consumers and carers were recruited primarily from outpatient clinics and related support groups (including those for survivors of the Wenchuan earthquake). The members of this panel also needed to meet the following criteria:

1. 18 years of age or above;
2. High school education or above;
3. Have had a lived experience of extreme distress following a potentially traumatic event, feels well enough to participate; Or have the experience of caring for someone who has experienced a potentially traumatic event, e.g. a member of a consumer advisory or advocacy group, providing peer support to others, etc.

To maximize the diversity of the study sample and the validity of the Delphi survey, we aimed to recruit at least 30 members for each panel. When the panel members received the invitation by email or WeChat, they could voluntarily choose whether or not to participate. The panel members were reimbursed100 RMB retail vouchers for completing at least two survey rounds.

### Data collection and analysis

When participants entered the questionnaire via a link in the invitation, they were asked to rate the importance of each statement for inclusion in the guidelines to help those who have experienced a potentially traumatic event in China. Each statement was rated using a five-point scale with the following options: *Very important, Important, Unclear, Unimportant, Very unimportant*. Statements were immediately included in the guidelines if they were endorsed by ≥80% of both panels as either very important or important. Statements were re-rated in the subsequent round if they were endorsed by ≥80% of one of the panels and by 70.0–79.9% of the other or by 70.0–79.9% of both panels. Statements were immediately excluded if they were rated as very important or important by less than 70% of at least one panel. Each statement could only be re-rated once.

A total of three survey rounds were conducted. Following each of the three rounds, panel members were presented with a brief summary of the results from the previous round, listing items to be included, re-rated, or excluded. In Round 1, there was space at the end of each section for panel members to add comments for that section, as well as to suggest new statements that had not been included. These comments and suggestions made by the panel members in Round 1 were carefully reviewed by the working group and used to develop new items for Round 2. Suggestions and statements were accepted and added to Round 2 if they were clear actions and presented a novel idea. Suggestions were rejected if they were covered by statements in the existing questionnaire, if they were too vague and general, or were more appropriate to clinical treatment than first aid.

The Round 3 questionnaire comprised statements that were presented in Round 2 for the first time and that required re-rating in a further round. Statements that still did not achieve consensus after three rounds were rejected from inclusion in the guidelines.

The correlation between the statement endorsement rates from the two panels was measured by Pearson’s correlation coefficient using the SPSS software (version 17.0).

### Guidelines development

All statements endorsed as either “Very important” or “Important” by ≥80% of both panels were included into the final guidelines. The first author drafted the guidelines by writing the list of endorsed statements into sections of prose based on common themes. Where possible, statements were combined and repetition deleted. The draft guidelines were then presented to the Chinese working group for structural and linguistic polishing to make them more easily understood by the general public in China. After several drafting iterations, the final draft of guidelines was formed, a copy of which was sent to each panel member for review. They could provide feedback on the structure and wording of the guidelines to make the text clearer. The final guidelines are provided in the online supplementary material.

## Results

### Participants

A total of 63 panel members (30.2% male) completed the Round 1 survey, including 32 mental health professionals and 31 consumers and carers. Their basic demographic information is provided in Table 1. The average age of panel members was 37.0 years (SD = 8.7, range 20–62), and 95.2% of them had university education or above.

**Table 1:** Demographic characteristics of participants in the Round 1 survey

Characteristic		Panel of mental health professionals (***n*** = 32)	Panel of consumers and carers (***n*** = 31)	All (***n*** = 63)
**Sex N (%)**	Male	11 (34.4)	8 (25.8)	19 (30.2)
	Female	21 (65.6)	23 (74.2)	44 (69.8)
**Age (years)**	Range	27 ~ 62	20 ~ 50	20 ~ 62
	Mean ± sd	39.2 ± 9.0	34.9 ± 7.9	37.0 ± 8.7
	Median	38	32	36
**Education N (%)**	Senior high school	0 (0)	3 (9.7)	3 (4.8)
	College/university	14 (43.8)	24 (77.4)	38 (60.3)
	Postgraduate or above	18 (56.2)	4 (12.9)	22 (34.9)
**Years of relevant experience**	Range	2 ~ 30	0 ~ 22	0 ~ 30
	Mean ± sd	9.2 ± 7.3	5.7 ± 5.0	7.5 ± 6.5
	Median	9	5	5

The 32 mental health professionals included 13 psychiatrists, 11 psychotherapists, 5 psychology teachers (who are also counsellors) and 1 mental health social worker. The lived experience panel included 5 members of people with experience of traumatic events and 26 carers.

Table 2 shows the continuity of participation across the three rounds. In the end, 92.1% (*n* = 58) of the participants completed all three rounds of the survey. Non-responders were contacted to remind them to complete the questionnaire up to three times. Some (7.9%) didn’t complete the survey because they were too busy or lost touch.

**Table 2:** Participation of the two panels in each round

	Panel of mental health professionals	Panel of consumers and carers	All
Round 1	32	31	63
Round 2 (Retention rate over 2 rounds)	31 (96.9%)	30 (96.8%)	62 (98.4%)
Round 3 (Retention rate over 3 rounds)	31 (96.9%)	27 (87.1%)	58 (92.1%)

### Ratings of the statements

**Fig. 1:**
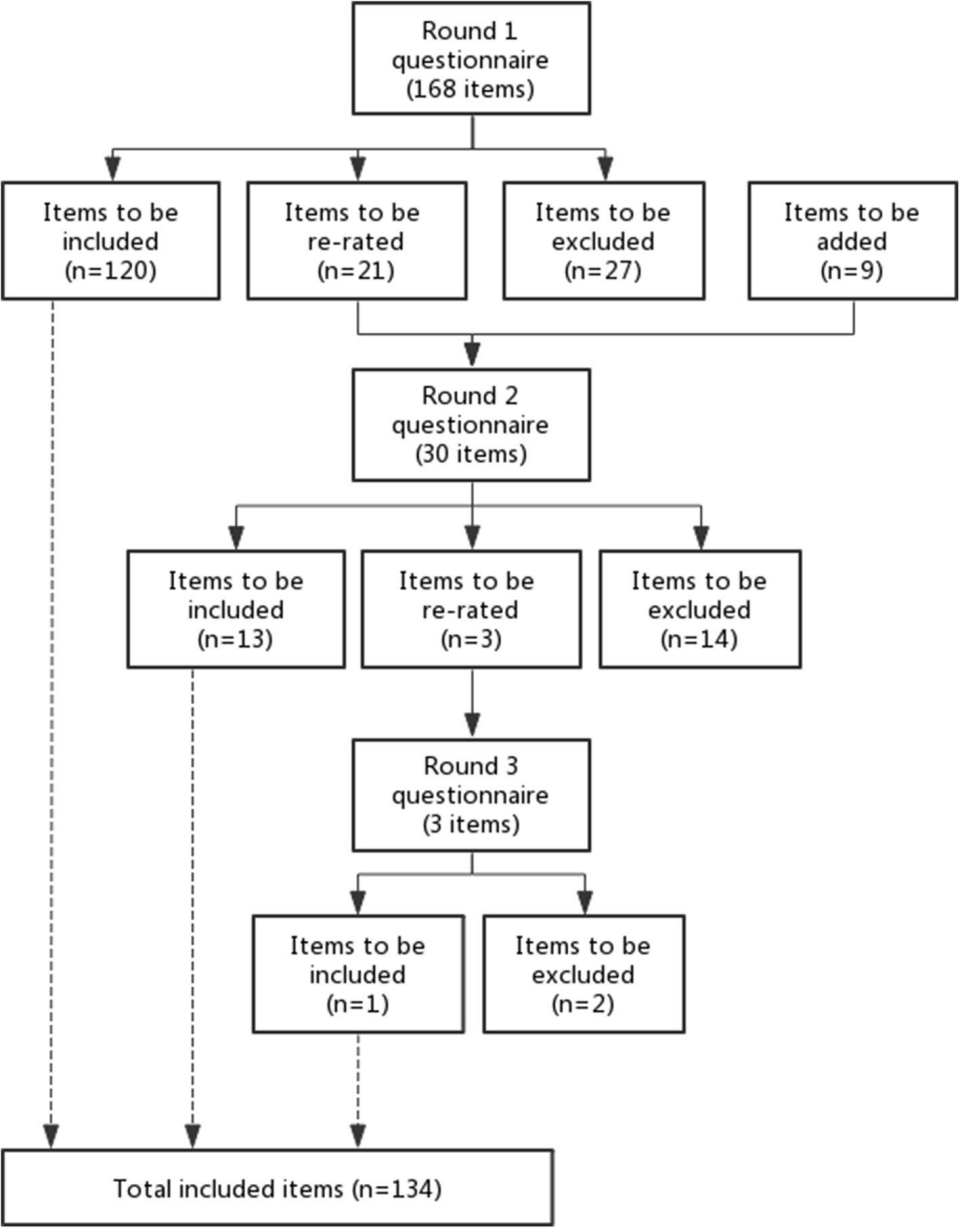
Overview of statements throughout the 3 rounds of questionnaires

An overview of the three rounds of the Delphi study is provided in Fig. 1. The Round 1 questionnaire contained 168 statements and 9 new statements were developed from the comments of the panel members in Round 1, resulting in 177 statements being rated across the three rounds. After the three survey rounds, 134 (75.7%) statements were endorsed for inclusion in the final guidelines: 120 statements were endorsed in Round 1, 13 in Round 2 and 1 in Round 3. A total of 43 (24.3%) statements were not endorsed for the guidelines: 27 statements were rejected in Round 1, 14 in Round 2 and 2 in Round 3 (see Additional file 1 for a list of all the statements and their respective rates of endorsement).

One section in the Round 1 questionnaire was about “Providing support in the weeks and months following a potentially traumatic event”. Of this section, 9 statements were about how long the effects of a potentially traumatic event last before the first aider should encourage the person to seek professional help. For example: “*The first aider should encourage the person to seek professional help if the post-trauma symptoms are interfering with their usual activities for: 1)2 weeks or more, 2) 4 weeks or more.”* Participants were required to rate the importance of each option in the statement. In Round 1 survey, the “*4 weeks or more*” option for each statement was endorsed by 80% or more of each panel as either very important or important. Therefore, after discussion, we directly included the “*4 weeks or more*” option for all 9 statements, which was also consistent with the English-language guidelines. The “*2 weeks or more*” option was excluded directly and not re-rated in the Round 2 survey.

The endorsement rates from the professional panel and the lived experience panel were significantly correlated (*r* = .68, *p* < .001) in Round 1, but some differences were also identified. One statement with the greatest difference (29.3%) in endorsement rates between the professional panel (90.6%) and the lived experience panel (61.3%) was “*If the person wants to talk about the potentially traumatic event but this is too distressing for the first aider, they should find someone else for the person to talk to*” After discussion, the Chinese working group agreed that this action was important in the first aid work, so we didn’t reject it directly. Instead, we revised the statement by adding examples to clarify ‘*someone else (e.g. friends, other first aiders or professionals’)* and included it in Round 2 for re-rating. However, in the second round, this statement was excluded because the endorsement rate of the lived experience panel still didn’t reach 80%.

### Differences in statements between the Chinese guidelines and the English-language guidelines

As summarized in Table 3, there were some differences in the endorsement of statements between the English-language and Chinese guideline studies. Thirty-three statements that were rejected in the redevelopment of the guidelines for English-speaking countries, but were endorsed in the current study, and 8 statements were endorsed in the English-language Delphi, but rejected in current study. In addition, 6 new statements developed from the comments in the Round 1 questionnaire were added in the guidelines for China.

**Table 3:** Changes in the adapted mental health first aid guidelines of trauma for China

Changes(n)	Statements
***Section 1. Background information(6 statements in the English version)***	
**Added (new statements developed based on comments from panellists,** ***n*** **= 2)**	The first aider should be aware that for most people, their responses to a potentially traumatic event may be “normal reactions in an abnormal situation”, do not necessarily mean “being mentally ill”.
	The first aider should be aware that they can also be influenced by the potentially traumatic event.
***Section 2. Actions to be taken at the site of a potentially traumatic event(39 statements in the English version)***	
**Endorsed (statements endorsed for inclusion in the Chinese guidelines but rejected in the English-language guidelines,** ***n*** **= 7)**	The first aider should find out what the person’s immediate needs are (e.g. food, clothing, shelter, medical help or emotional support) and attempt to meet them.
	The first aider should try to minimise the person’s exposure to potentially upsetting sights and sounds, e.g. injured people or flashing lights.
	If the person has been a victim of crime, the first aider should consider the possibility that forensic evidence may need to be collected (e.g. evidence on clothing or skin) and should encourage the person to preserve such evidence.
	The first aider should discourage the person from making any impulsive decisions because they may not be thinking clearly.
	If the person’s loved ones or friends are not present, the first aider should offer to contact them.
	If the person has been separated from loved ones during the potentially traumatic event and wants to be reconnected with them, the first aider should try to help them do so.
	If the person is asking for information, the first aider should find out from professional helpers what information they are allowed to pass on to the person.
**Added (*n*** **= 2)**	If the person shows aggressive behaviors like suicide and self-harm, the first aider should try to keep calm and try to comfort the person.
	If the person hurts someone or destroy something, the first aider should ensure his own safety and then call the police or a professional to help.
***Section 3. Talking about the trauma(53 statements in the English version)***	
**Endorsed (*n*** **= 8)**	If the person begins a sensitive conversation and the first aider does not think it is the ideal place to talk to the person, the first aider should suggest finding an environment likely to be safe, comforting and free of distractions.
	The first aider should encourage the person to talk about their feelings, but only if the person feels ready to do so.
	If the person talks repetitively about the potentially traumatic event, the first aider should listen.
	The first aider should tell the person that what happened was not their fault, but only if they know this to be true.
	The first aider should not avoid talking about the person’s experiences.
	If the person wants to tell their whole story about the potentially traumatic event, the first aider should give the person enough time to do so.
	If the person seems to be ‘spaced out’, ‘shuts down’ or is struggling to communicate, the first aider should encourage the person to move a little, e.g. change their posture, do some stretches.
	If the person seems to be ‘spaced out’, ‘shuts down’ or is struggling to communicate, the first aider should offer to talk to the person at another time.
**Added (*n*** **= 1)**	The first aider should remind the person that there are people who care about him and love him.
**Excluded (statements rejected for inclusion in the Chinese guidelines but endorsed in the English-language guidelines,** ***n*** **= 3)**	The first aider should not probe for details of the potentially traumatic event.
	The first aider should not offer religious solace by saying things like “God has reasons”.
	If the person wants to talk about the potentially traumatic event but this is too distressing for the first aider, they should find someone else for the person to talk to.
***Section 4. Providing support in the weeks & months following a potentially traumatic event(38 statements in the English version)***	
**Endorsed (*n*** **= 5)**	The first aider should discourage the person from making any major life decisions or big life changes, if at all possible.
	The first aider should encourage the person to talk about how the trauma has affected their relationships.
	The first aider should encourage the person to share their reactions with people who they think will be supportive.
	The first aider should encourage the person to share their memories with people who they think will be supportive.
	The first aider should encourage the person to seek help from a professional who treats people who have experienced trauma.
**Added (*n*** **= 1)**	If the person appears to suddenly return to their normal routine and behaviours after experiencing the trauma, you should still continue to keep an eye on them.
**Excluded (*n*** **= 2)**	The first aider should tell the person about any sources of information available for survivors, e.g. information sessions, fact sheets and phone numbers for information lines.
	The first aider should encourage the person to seek professional help if they misuse alcohol or other drugs to deal with the trauma at any time.
***Section 5. Experiences of abuse(36 statements in the English version)***	
**Endorsed (*n*** **= 10)**	If the person discloses any abuse associated with criminal activity, the first aider should report it to the police, if it is safe to do so.
	If the person discloses any abuse associated with criminal activity, the first aider should encourage the person to report it to the police, if it is safe to do so.
	If the person discloses that they are being abused, the first aider should encourage the person to tell the perpetrator to stop, if it is safe to do so.
	If the person discloses that they are being abused, the first aider should encourage the person to tell the perpetrator how it is affecting them, if it is safe to do so.
	If the first aider is concerned that the person is at risk of harm from someone else, they should offer to call the police and report the situation.
	If the first aider is concerned that the person is at risk of harm from someone else, they should encourage the person to call the police and report the situation.
	If the first aider is concerned that the person is at risk of harm from someone else, they should offer to call an appropriate helpline on behalf of the person, e.g. family violence helpline.
	If the person asks the first aider not to tell anyone about the abuse they have experienced, the first aider should respect their wishes, unless they are at risk of immediate harm.
	If the person asks the first aider not to tell anyone about the abuse they have experienced, the first aider should respect their wishes, unless the abuse is likely to be a criminal offence.
	If the person discloses abuse that happened in the past, the first aider should tell the person that they believe them.
**Excluded (*n*** **= 3)**	If the person discloses abuse, the first aider should listen to them and not feel that they have to provide solutions or advice.
	If the first aider expresses their concerns about signs of physical abuse and the person dismisses them and becomes angry, the first aider should explain they only asked out of concern and will continue to be there if the person needs someone to talk to.
	If the person begins to relate details of the abuse that the first aider finds distressing, the first aider should ask the person if they would like assistance finding someone else they can talk to.
***Section 6. Adolescents(11 statements in the English version)***	
**Endorsed (*n*** **= 3)**	If appropriate to the relationship, the first aider should contact the adolescent’s school about any additional support they may need.
	The first aider should encourage the adolescent to use face-to-face contact with friends rather than through social media.
	If the adolescent does not want to talk about what has occurred, the first aider should encourage the adolescent to talk about their feelings rather than about what has occurred.

Compared to the English-language guidelines, notable differences were seen in section 2 “*Actions to be taken at the site of a potentially traumatic event*”, with Chinese experts more likely to endorse statements more relevant in cases of natural disasters such as earthquakes and floods, including items about finding out about immediate living, medical and emotional needs, help with contacting loved ones and finding out from professionals what information to pass on. Safety was emphasized both for the person at risk and the mental health first aiders across the sections. More statements about encouraging social support were endorsed. These included those related to sharing how the trauma affected relationships, reactions and memories with supportive people, and obtaining additional support from school and face to face contact with friends in the case of adolescents.

## Discussion

This Delphi expert consensus study aimed to develop guidelines for members of the public in China providing mental health first aid to people affected by a traumatic event. This was achieved by engaging Chinese professionals involved with providing help and support to people following traumatic events and people who had traumatic experiences or their carers. The results show some key similarities and also some important differences between the Chinese and English-language guidelines.

### Comparison between statements in the guidelines for China and for English-speaking countries

There were 31 additional statements in the final Chinese guidelines compared to the English-language guidelines. These additional statements included many specific actions that first aiders should take, such as trying to meet the person’s immediate needs, talking to them about the trauma and helping them to deal with issues related to abuse. In the section on “*Actions to be taken at the site of a potentially traumatic event*”, the statement “*if the person has been a victim of crime, the first aider should consider the possibility that forensic evidence may need to be collected (e.g. evidence on clothing or skin) and should encourage the person to preserve such evidence*” may reflect the panels’ expectation that first aiders should play an important role and provide more practical help. In addition, two statements related to recommendations that the first aiders should contact the family members or friends of the person who had experienced a potentially traumatic event. This may show the importance of the support of family and friends in the Chinese context. While people in English-speaking countries may be more likely rely on public services (e.g., doctors, crisis intervention teams, mental health professionals), these are less widely available in China [40–42]. In the section on “*Experiences of abuse*”, four additional statements related to asking the police for help were included. “*If the person discloses any abuse associated with criminal activity*” or “*if the first aider is concerned that the person is at risk of harm from someone else*”, the first aider should (or encourage the person) to report it to the police. It may be that, compared to English-speaking panelists, Chinese health professionals and consumers saw this as less likely to be common practice and therefore as needing to be emphasised in the guidelines. However, these items may also reflect that fact that there are few other organizations or resources in China that can provide help to people who have experienced abuse, or that they are not very effective [43].

However, as in the English-language guidelines, these new Chinese guidelines emphasize that first aiders are not professionals and they should not be a substitute for professional help. Inappropriate intervention by a non-professional may not only fail to help a person, but may also have a negative impact on the person concerned and the first aider themselves [44]. Therefore, an additional statement was added to the guidelines “*The first aider should be aware that they can also be influenced by the potentially traumatic event*”, emphasizing that first aiders need to be mindful of their own mental health in order to prevent vicarious trauma. This view may be informed by China’s previous experience in psychological first aid [45]. Previous studies have shown that volunteers who have received disaster assistance training have lower levels of vicarious trauma than those who have not. Student volunteers, media workers and other aid volunteers also reported having higher levels of vicarious trauma than mental health professionals [45].

### Comparison between ratings of professional and lived experience panels

Although the professional panel and lived experience panel agreed on most of the statements, they differed (difference in the endorsement rates > ±10%) on some of the statements. For example, the lived experience panel rejected the statement ‘*If the person wants to talk about the potentially traumatic event but this is too distressing for the first aider, they should find someone else for the person to talk to*’ (professional: 90.6%; lived experience: 61.3%) in Round 1. It is possible that the lived experience panel members thought it important to have the first aider’s company and were reluctant to be separated from them to speak to others or they might think they couldn’t talk to anyone else if they couldn’t talk to the first aider. In addition, 90.6% of the professional panel members endorsed that ‘*If the person does not wish to talk to the first aider about how they are feeling, they should encourage the person to consider calling a help line or using other community resources*’, but only 71.0% of consumers and carers rated this statement as either very important or important in Round 1 (87.1% vs. 76.6% in Round 2). But the two panels were more in agreement (93.8% vs. 96.8%) on another statement ‘*The first aider should encourage the person to talk about their feelings, but only if the person feels ready to do so*’. These differences may reflect the desire of a person who has experienced a traumatic event for their wishes to be respected. If the person is unwilling to talk, then the first aider should not push them to talk, instead they should wait or offer other help until the person is ready to talk. Similarly, the professional panel rejected (endorsement rate 56.3%) the statement ‘*If the person begins to relate details of the abuse that the first aider finds distressing, the first aider should protect themselves by encouraging the person to talk about other things’*, while 80.6% of consumers and carers endorsed it. The professional panel may consider that first aiders should care for their own emotions, but the consumers and carers may want their experiences and feelings to be fully respected and understood and not to be interrupted.

### Limitations and strengths

Several limitations of this study should be considered. Firstly, only five people with traumatic experiences were included in the lived experience panel. It was difficult to recruit people with traumatic experiences (including, for example, families who had lost their only child), as they were unwilling to participate for fear of recalling their loss. It may also be because this survey was mainly conducted online. In China, many people with mental disorders and their families, or those who have experienced trauma, feel stigmatized for their experiences. For this reason, they may be reluctant to reveal these private experiences to others, particularly in an online survey [46–48]. In China, most mental-health-related surveys have been conducted face to face, offering respondents with concerns the opportunity to ask questions of an interviewer in person. As it is relatively easy to refuse to participate or drop out of online surveys, face-to-face surveys may increase participation through better explanation of and provision of care and support to people with lived experience. Future studies could use face-to-face interviews to explore these issues. Secondly, most of the participants in this study were recruited from urban areas (mainly from Shanghai and Zigong), which may limit the generalizability of the findings. Due to the diversity of different regions in China, the uneven distribution of mental health resources between urban and rural areas and in the eastern and western regions of China [49], and the wide variation in education levels, the applicability of the guidelines in rural areas and for people with limited education requires further study.

On the other hand, the study has several strengths. Firstly, the number of members of the two panels (32 professionals and 31 consumers and carers), met the minimum panel size of 20 for a Delphi study [27], which ensured the reliability of the results. In addition, the drop-out rate of participants in the second and third rounds was lower than in many previous Delphi studies [29, 34, 50], possibly because experts were more interested in trauma issues. The third round of the survey was conducted during the COVID-19 outbreak when many people suffered from anxiety, stress and other mental problems, therefore, the focus on trauma may have improved engagement in the study. The third round of surveys also included links to reimbursement for participants, which may also have boosted participation.

### Important considerations when using the guidelines in China

This study reports on the development of the guidelines for assisting a person affected by a potentially traumatic event in China. For the guidelines to have beneficial impact, they will need to be effectively disseminated and incorporated into practice. The guidelines may be disseminated as a standalone project and will also be used to inform the development of a MHFA training course for China. Both these activities require consideration of the current state of China’s mental health system, resources, and policies. The Chinese government attaches increasing importance to psychological crisis intervention and psychological assistance, offering promising opportunities to develop and implement MHFA training. Policymakers may be encouraged to include MHFA training as part of plans to improve public mental health literacy.

In addition, the last section of the guidelines, which is about how to help an adolescent who has experienced a potentially traumatic event, is an area that has not been emphasized in the other Chinese mental health first aid guidelines (depression, suicide and psychosis) [34–36]. Consistent with the emphasis in this section, Chinese policy has called for a focus on adolescent mental health to address the lack of trauma interventions for adolescents in China [12, 13]. Many cases of adolescent abuse, school bullying or family violence go undetected, with appropriate interventions even less common [51, 52]. Therefore, help from the public is particularly important.

## Conclusions

Through the Delphi process, the Chinese mental health first aid guidelines for assisting a person affected by a potentially traumatic event have been developed to ensure they are current and include the most appropriate helping actions. While many of the statements are similar to the guidelines for English-speaking countries, the panelists adapted the guidelines to China’s situation, including more detailed actions on how to discuss trauma and how to help. These guidelines will be disseminated as standalone products and will also be used to form the basis of an MHFA training course aimed at educating the public in how to provide mental health first aid to a person who is experiencing a potentially traumatic event in China. Future work needs to evaluate their impact on the first aiders’ helping behaviors and on the recipients of the first aid, as far as this is possible. This will assist researchers to develop an evidence base for MHFA training in China.

## Supplementary Information

**Additional file 1.**

**Additional file 2.**

## Data Availability

The dataset used and analyzed during the current study are available from the corresponding author on reasonable request.
