# Supplementary material for: Development of Chinese mental health first aid guidelines for assisting a person affected by a traumatic event: a Delphi expert consensus study
Source: BMC Psychiatry. 2021 Dec 1;21:600. doi: 10.1186/s12888-021-03606-3 (PMC8633911; doi:10.1186/s12888-021-03606-3)
Supplement: Supplementary file 2 — Additional file 2. [file 12888_2021_3606_MOESM2_ESM.docx]

# 精神健康急救指南 – 创伤篇

## 指南的目的

指南旨在指导公众如何为可能正在经历潜在创伤事件的人（在本指南中称为“救助对象”或“对方”）提供初步帮助，即实施“精神健康急救”。

| **精神健康急救 (Mental Health First Aid, MHFA)**：为发生精神健康问题，或现有精神健康问题恶化，或正在经历精神健康危机（如自杀或创伤经历）的人提供**初步帮助**，直至对方获得适当的专业帮助或危机解除。 |
| --- |

所谓精神健康急救人员（后简称“急救人员”），即为经历精神病问题的人提供救助的非精神卫生专业人士，他们可以是救助对象的家人、朋友，也可以是同事或邻居，等等。急救人员的作用是为经历潜在创伤事件的人提供初步帮助，直至对方获得适当的专业帮助或危机解除。

## 指南的制定

指南的内容是在综合了经验丰富的精神健康领域专家、潜在创伤事件经历者及有经验的照料者意见的基础上制定的。

## 指南的使用

指南所提供的指导方针仅为一般性建议。每个救助对象的情况不同，急救人员需要根据对方的具体情况对所提供的帮助作出适当调整。

**什么是潜在创伤事件？**

潜在创伤事件是指一种强烈而痛苦的经历，可能对一个人的生命、身体或心理健康造成严重威胁，常见的有人际暴力（如家庭暴力、校园欺凌、网络暴力、虐待儿童、虐待老人、身体或性侵犯、抢劫）、意外事故（如交通事故或工作场所事故），以及目睹可怕的事情发生。集体创伤事件包括战争、虐待、恐怖袭击、大规模枪击和严重自然灾害（如洪水、地震、飓风、海啸和森林火灾）。

许多过去的和正在发生的事件都有可能造成创伤，但并非所有通常被认为是创伤的事件都会给人造成极大痛苦。突然回忆起过去的潜在创伤事件也可能引发创伤体验。间接暴露也会导致创伤，例如目睹他人经历创伤事件，知道他人发生了创伤事件，反复或过度暴露于创伤事件的细节中。

**潜在创伤事件后的反应**

作为急救人员，你应了解经历潜在创伤事件后最初的常见反应，也要知道每个人在经历潜在创伤事件后的反应会有很大不同，不应假定救助对象有任何特定的反应。你需要知道什么样的表现和症状意味着潜在创伤事件已经引起心理健康问题、可能引发的长期病症是什么（请参见下面文本框内容）。对大部分人而言，这些可能是“异常状况下的正常反应”，并不一定意味着“精神出了问题”。救助对象可能有作为幸存者的内疚感，觉得其他人死亡或受伤而自己幸免是不公平的。人们对潜在创伤事件的反应存在文化差异和个体差异，例如，有些人认为在陌生人面前表达脆弱或悲伤是不合适的。

| 人们在经历潜在创伤事件后，通常即刻便会感到非常痛苦，可表现为情绪紧张、焦虑增加、睡眠或食欲受到影响。其他反应可能包括悲伤、内疚、恐惧或愤怒，通常这些反应会在一个月内缓和下来，只有一部分人会发展为精神障碍。  **急性应激障碍和创伤后应激障碍^1^**  急性应激障碍和创伤后应激障碍的症状比较相似。一个主要的症状是创伤的再体验，可能的形式包括反复出现与创伤事件相关的梦、闪回、侵入性记忆或痛苦。出现回避行为，如持续回避与事件相关的事物（如人、地点、对话、活动、物体、情境），可能持续数月或数年。还有持续增加的情绪困扰症状（持续的警觉、惊跳反应、易受惊吓、易激惹、攻击性、失眠）。也可能过度责备自己或他人，对他人和外部世界的兴趣减退，并可能无法完全记住创伤事件。  如果一个人在创伤事件后的几天或几周内出现这些症状，就可能被诊断为急性应激障碍。当症状持续一个月或更长时间时，可能被诊断为创伤后应激障碍。急性应激障碍是一种短暂的应激反应，经历了急性应激障碍的人中只有一部分会继续发展为创伤后应激障碍。创伤后应激障碍也可能在创伤事件过去数月或数年后才发生。  *1改编自：美国精神医学学会（2013）.精神障碍诊断与统计手册（第5版）* |
| --- |

**在潜在创伤事件现场可以做些什么？**

**优先协助**

当你参与救助时，要意识到自己也可能会受到潜在创伤事件的影响。提供帮助前，你应首先考虑自己的情绪状态是否适合给他人提供支持。如果不适合，则应先考虑照顾自己，并尝试寻找其他可以为救助对象提供帮助的人。

在采取任何行动前，应首先确认接近救助对象是否安全，例如，是否存在火、武器或残骸造成的危险。根据情况联系适当的紧急援助，例如，救护车、警察、消防队。如需要，应为救助对象寻找医疗救助。请注意，你不应取代专业救援人员的角色，因为他们可能会更好地满足救助对象的迫切需要。

在采取行动前，应先观察救助对象正在接受何种帮助以及是否需要额外帮助。如果对方已经在接受帮助，可以询问提供帮助的人是否需要协助或休息。

如果已经有医疗或紧急服务专业人员在现场，你应该遵从现场专业救援人员的指示，不应在救助对象面前批评专业救援人员的工作。如果救助对象要求你提供信息，你应先咨询专业救援人员自己可以将哪些信息告知救助对象。

**如何协助**

如果你不认识救助对象，应向对方解释自己的职责以及为何在场。尽量不要显得很匆忙或不耐烦。尽量为救助对象创造一个安全的环境，例如，让其远离交通繁忙的地方，远离火或残骸等。弄清楚救助对象的迫切需求是什么（例如，食物、衣服、遮盖物、医疗救助或精神支持）并尽量满足。你应该意识到并顾及对方的舒适和尊严，例如，给对方提供可以遮盖身体的物品（如一条毯子），并让旁观者和媒体走开。尽量避免使救助对象暴露在可能令其不安的场景和声音中，例如，受伤的人或闪烁的灯光。如果救助对象看起来不知所措或犹豫不决，你应协助对方做出必要的决定（例如，关于安全的决定），还应阻止对方做出任何冲动的决定，因为对方可能没有考虑清楚。

关注救助对象的身体或精神状态，观察是否有变差的迹象，并做好为对方寻求紧急医疗帮助的准备。例如，一个看上去没有外伤的人可能会有内伤，而这些内伤会慢慢显露出来。

尽量让救助对象了解当前正在发生的以及接下来可能会发生的事情。为对方提供准确的信息，或按照实际情况承认自己缺少相关信息。

如果救助对象的亲人或朋友不在现场，你应主动向对方提出联系他们。如果救助对象在潜在创伤事件中与亲人分开，并希望能与他们重新取得联系，你应尽可能帮助对方达成心愿。不要做出任何你可能无法兑现的承诺，例如，“我马上送你回家”。

如果你认为有人不是为了维护救助对象的最佳利益而行事（例如，不合时宜的媒体采访或阻止救助对象向官方举报），你应尽量保护救助对象。如果救助对象是犯罪活动受害人，你应该考虑到可能需要收集法医证据（例如，留在衣服或皮肤上的证据）并鼓励对方保留这些证据。

如果救助对象做出自伤、自杀等过激行为，你应尽量保持冷静，并尝试安抚救助对象。（参见《精神健康急救指南-自杀篇》）如果对方有伤人毁物的过激行为，你应首先确保自身安全，然后请求警察或专业人员协助处理。

**如何与经历潜在创伤事件的人交谈?**

应与救助对象平等沟通，而不是以一个专家的姿态居高临下。不管救助对象的情绪状态如何，与其交谈时应尽量保持冷静。即使对方可能无法很好地沟通，例如，重复说过的话、语速缓慢或吐字不清，也应表现出耐心，要让救助对象感受到自己正在倾听。如果救助对象开始谈论敏感话题，但你认为当下的环境并非理想的交谈场所，应建议寻找一个安全、舒适且不会被打扰的环境进行交谈。

在救助对象感到准备好了的时候，应鼓励他谈论自己的感受，但不应告诉他应该有什么感觉。避免阻止对方表达自己的感受，例如说，“不要哭”或者“冷静”。不要跟救助对象说“振作起来”、“向前看”或者“往好的方面想”，因为潜在创伤事件的影响不是单靠他个人的意志力就能克服的。应向救助对象表示，在这种情况下他的反应是很常见的，以使其安心。另外，应提醒救助对象还有人在关心他、在爱他。

**谈论救助对象的经历**

如果救助对象想要讲述关于潜在创伤事件的整个过程，应给予他足够的时间。救助对象也可能不记得潜在创伤事件的所有细节。如果救助对象重复谈论该事件，你应该认真倾听。

如果可以确定救助对象在此事件中没有过错，应明确告诉对方所发生的事情并不是他的错。如果救助对象对自己在潜在创伤事件中的反应感到羞愧或内疚，你应该宽慰对方：“在这种情况下人们不会有意识地选择怎样应对，因为这些反应通常是自动和本能的。 ”如果救助对象开始哭泣或努力忍住不哭，应告诉对方可以通过哭泣来宣泄自己的情绪，并表达当下的任何感受。

不要害怕承认自己不知道说什么。如果救助对象把你拒之门外，你应耐心询问是否能以其他方式帮到他。

| **不要做什么**   - 不要暗示所发生的事情是救助对象的错，或暗示对方应该在潜在创伤事件发生时做出不同的反应或采取不同的行动。 - 不要回避谈论救助对象的经历。 - 不要强迫救助对象讲述潜在创伤事件或其对事件的感受。 - 不要对救助对象的经历轻描淡写，例如，“情况本来可能更糟糕”，“你会没事的”或“你现在应该已经恢复了”。 - 不要打断救助对象然后分享你自己的感受和经历。 - 不要把救助对象的创伤经历与其他人的经历相比较。 |
| --- |

**应对谈话过程中的挑战**

诸如退缩、易怒和脾气差等行为可能是一个人对潜在创伤事件的反应，尽量不要认为这些行为是针对你的。即使你觉得对方的行为有些挑衅，也应尽量去关心他。

如果感觉救助对象因为谈话而感到痛苦，应询问他是否需要休息一下。如果发现对方在“走神”，出现脑子“短路”现象，或为沟通发愁，不要认为他一定是不想交谈，可以鼓励他稍微活动一下（如，改变一下姿势，做一些伸展运动），询问他此时此刻有什么需要。不要强迫他谈论令其痛苦的事情，可以提议在其他对方认为合适的时间与之交谈。

如果救助对象出现潜在创伤事件的情景再现，你应该提醒对方此刻他是安全的，并引导他关注此时此地，例如，关注周围的环境。避免突然移动或做出任何可能惊吓到对方的事情，并询问对方当潜在创伤事件情景再现时他希望得到怎样的帮助。

**如果救助对象遭受虐待，该怎么做？**

如果救助对象透露自己正遭受虐待，在安全的情况下，你应鼓励对方告诉施虐者停止这种行为，并让施虐者知道这种行为对他所造成的伤害。如果救助对象透露自己过去曾经受到的虐待，你应该告诉对方自己相信他，不要表露出“难以置信”的态度，并感谢对方能够信任并告诉自己。你应意识到经历过虐待的人可能不会轻易相信别人并且可能不愿意寻求其他人的帮助。如果救助对象开始提及让你感到不适的虐待细节，你应该从保护自己的角度出发，鼓励对方与专业援助组织交谈，而不是继续与你谈下去。

如果你看到了救助对象被虐待的身体痕迹（例如，瘀伤的新痕旧迹），你应与对方讨论自己的担忧。也许他并不理会你的担忧，但这不意味着你的担忧是错的。如果对方对你的担忧置之不理，而之后你又发现虐待的迹象，则应再次向对方提出自己的担忧。

如果你担心救助对象有被其他人伤害的危险，你应与他合作，确定接下来应该采取哪些措施来确保他的安全。提供能够确保对方安全的方法，例如，帮他寻找其他住所。协助对方确认其他可提供帮助的人。鼓励救助对象拨打合适的求助热线（如反家暴热线）或报警，根据对方的客观情况，也可以提议自己代替对方拨打这个电话。不要做任何会让你陷入危险的事情。

如果救助对象要求你不要告诉任何人其所经历的虐待，你应尊重对方的意愿，除非对方有立刻遭到重大伤害的危险或该虐待可能构成刑事犯罪。如果出于安全考虑，你认为有必要向其他人分享任何救助对象告诉你的信息，你应事先充分考虑这样做可能会对救助对象造成的影响，例如，人身安全或人际关系的影响等，并应征得对方同意。

你应该了解当地所有关于强制性举报的法律法规。如果救助对象透露任何自己经历的涉及犯罪活动的虐待，在确保安全的情况下，你应鼓励对方向公安机关报案或代替其报案，并鼓励对方向适当的援助组织寻求帮助。

**如何在潜在创伤事件发生后的数周和数月内提供帮助？**

**支持和理解**

即使在潜在创伤事件发生后数周或数月，救助对象的状况仍可能会时好时坏，而且并没有时间规律可以用来预测将会发生什么。应告知对方，每个人处理潜在创伤事件的节奏不同，但他一定可以从潜在创伤事件中慢慢恢复。如果可能的话，应避免让救助对象在此时做出任何重大的人生决定或改变。如果救助对象突然表现得一切正常，像从未经历过伤害，这有可能就是一种反常行为，不应掉以轻心。

救助对象可能会对别人觉得微不足道的事情或压力表现得更敏感，有许多事情可以促使他想起创伤事件并感到悲伤，他可能会突然记起事件的细节。创伤事件的周年纪念和媒体对类似事件的报道，都有可能使他想起该事件，在这些时刻，救助对象可能需要更多额外的支持。

**你应了解救助对象曾使用过的自助方法及其效果。**

鼓励对方与他认为能给予支持的人分享自己的反应和记忆，鼓励其谈论创伤事件是如何影响他的人际关系的，鼓励他使用那些对其潜在创伤事件可能会有帮助的社会支持服务，例如，妇联、亲友会。

**寻求专业帮助**

你应了解有哪些可以提供给经历创伤者的专业帮助，并鼓励对方主动寻求创伤治疗专业人员的帮助。

如果创伤事件发生4周或更长时间后，对方仍有以下表现，应鼓励其寻求专业帮助：

- 感觉非常心烦或害怕
- 感觉自己变得神经质、做噩梦
- 无法摆脱持续存在的强烈痛苦感
- 一直想着创伤事件
- 因创伤而完全无法享受生活
- 创伤后的症状干扰日常活动
- 行为非常不同
- 重要人际关系因创伤而受到影响（例如，疏远家人或朋友）
- 通过酗酒或滥用药物来应对创伤（参见《精神健康急救指南-问题饮酒篇》）

**青少年需要什么样的额外支持？**

青少年与成年人对潜在创伤事件的反应方式不同。如果你没有与青少年长期相处的经验，则应询问对方是否有可信赖的成年人来支持他。如果没有，应当帮他联系合适的服务，例如，未成年人或青少年心理辅导中心、学校或社区的心理服务、求助热线等。

如果你的身份合适，应联系对方所在的学校，获取可能需要的额外支持。鼓励其与朋友面对面接触，而不是通过社交媒体。

如果对方不想讨论发生了什么，应该让其知道你已做好随时与他交谈的准备；也可以询问对方是否有其他愿意交谈的对象。鼓励他表达自己的感受而不是发生了什么。
